# Neoplasms in the Nasal Cavity Identified and Tracked with an Artificial Intelligence-Assisted Nasal Endoscopic Diagnostic System

**DOI:** 10.3390/bioengineering12010010

**Published:** 2024-12-25

**Authors:** Xiayue Xu, Boxiang Yun, Yumin Zhao, Ling Jin, Yanning Zong, Guanzhen Yu, Chuanliang Zhao, Kai Fan, Xiaolin Zhang, Shiwang Tan, Zimu Zhang, Yan Wang, Qingli Li, Shaoqing Yu

**Affiliations:** 1 Department of Otolaryngology and Neck Surgery, Tongji Hospital, School of Medicine, Tongji University, Shanghai 200065, Chinaqiaoshanqian@ailiyun.com (G.Y.);; 2 Department of Allergy, Tongji Hospital, School of Medicine, Tongji University, Shanghai 200065, China; 3 Shanghai Key Laboratory of Multidimensional Information Processing, East China Normal University, Shanghai 200241, China; 4 Laboratory of Digital Health and Artificial Intelligence, Zhejiang Digital Content Research Institute, Shaoxing 312000, China

**Keywords:** computer-assisted surgery, nasal cavity, artificial intelligence, diagnosis

## Abstract

Objective: We aim to construct an artificial intelligence (AI)-assisted nasal endoscopy diagnostic system capable of preliminary differentiation and identification of nasal neoplasia properties, as well as intraoperative tracking, providing an important basis for nasal endoscopic surgery. Methods: We retrospectively analyzed 1050 video data of nasal endoscopic surgeries involving four types of nasal neoplasms. Using Deep Snake, U-Net, and Att-Res2-UNet, we developed a nasal neoplastic detection network based on endoscopic images. After deep learning, the optimal network was selected as the initialization model and trained to optimize the SiamMask online tracking algorithm. Results: The Att-Res2-UNet network demonstrated the highest accuracy and precision, with the most accurate recognition results. The overall accuracy of the model established by us achieved an overall accuracy similar to that of residents (0.9707 ± 0.00984), while slightly lower than that of rhinologists (0.9790 ± 0.00348). SiamMask’s segmentation range was consistent with rhinologists, with a 99% compliance rate and a neoplasm probability value ≥ 0.5. Conclusions: This study successfully established an AI-assisted nasal endoscopic diagnostic system that can preliminarily identify nasal neoplasms from endoscopic images and automatically track them in real time during surgery, enhancing the efficiency of endoscopic diagnosis and surgery.

## 1. Introduction

After its initiation in the 1980s, nasal endoscopy has been increasingly used for the clinical diagnosis of nasal disease. As the techniques of endoscopy continued to develop, it was also used as an auxiliary tool for nasal surgery [1]. Consequently, the procedure of identifying diseases through endoscopic examination holds significant importance.

Neoplasms of the nasal cavity can be detected early and removed by nasal endoscopic surgery. Although tumors possess certain characteristic features, such as surface ulceration and irregular shape, they cannot be easily distinguished from other non-tumorous neoplasms of the nasal cavity, such as inflammatory nasal polyps (NPs) and mycotic masses formed by fungal infections. Nasal inverted papilloma (NIP) is the most common tumor of the nasal cavity; it cannot be easily distinguished from polyps and can be occasionally misdiagnosed by experienced clinicians, whereas other neoplasms, especially malignant neoplasms, do not have a similar phenotype and can present in various shapes such as cauliflower-like, ulcerated, etc. With rapid advances in imaging technologies and radiotherapy, the local control rate for patients with early-stage nasal malignancies has increased to 95%. Hence, early detection is crucial for enhancing the overall survival rate of patients with nasal malignancies [2]. The surgical approach, resection extent, and prognostic management vary among different neoplasms [3]. That is why we need to distinguish these lesions. Traditionally, neoplasms are diagnosed through pathology, a process that occurs postoperatively and offers no intraoperative guidance. Furthermore, pathological assessments are confined to resected tissue and are unable to delineate the boundaries between neoplasms and normal tissue [4]. Consequently, there is an urgent clinical demand for an endoscopy-assisted diagnostic system capable of accurately classifying and diagnosing neoplasms under endoscopic vision, while also guiding the extent of surgery in real-time.

Artificial intelligence (AI) represents a remarkable advancement stemming from the rapid evolution of computer technology. Deep learning techniques have been developed and integrated into medical treatment, providing invaluable assistance to clinicians. Recent studies have demonstrated that deep learning outperforms experts in the application of medical visual tasks [5,6,7]. In particular, convolutional neural networks (CNNs) have exhibited excellent performance in medical image classification [8,9,10]. Based on this, research has been conducted on AI tracking systems, such as SiamMask, which is an innovative tracking and segmentation technique [11]. This technique can locate the tracked targets to both assist the diagnosis of lesions and indicate their extent during surgery [12].

AI has been used for nasal endoscopy-assisted diagnosis of NIPs with high accuracy rates; however, the existing study was confined to a single type of nasal neoplasm [13,14]. To enhance the nasal endoscopy’s capacity for automatically diagnosing multiple types of nasal neoplasms, this study developed an AI-assisted nasal endoscopic diagnosis system by collecting image data of four types of nasal neoplasms and analyzing the accuracy rate by comparing them with data from experts and pathological diagnoses. The autonomous learning of the system is expected to enable the accurate diagnosis of neoplasm types by nasal endoscopy. Additionally, the system will enable real-time intraoperative tracking of the extent of lesions using SiamMask tracking technology. This study comprehensively evaluated the ability of AI technology in assisting nasal endoscopic diagnosis and treatment.

## 2. Materials and Methods

### 2.1. Preparation of Clinical Data and Nasal Endoscopic Image Data

A total of 1050 surgical videos of nasal patients in Shanghai Tongji Hospital from March 2020 to July 2021 were selected (with one patient corresponding to one video) to establish a target segmentation algorithm for the neoplasms of the nasal cavity. Additionally, we gathered surgical videos of 215 nasal patients from Tongji Hospital between July 2021 and March 2022 as a prediction set (independent of the training, validation, and test sets) to compare the diagnostic performance between the SiamMask model and clinical visual assessment by human experts.

Each patient had a definitive pathological diagnosis. Based on standardized clinical records, we collected clinical data for each patient, including gender, age, nasal obstruction, epistaxis, hyposmia, and other relevant information. The 1050 videos previously collected were randomly divided into the training dataset (630 cases), validation dataset (210 cases) and testing dataset (210 cases) in a ratio of 3:1:1. The 215 videos subsequently collected were all used as the prediction set. At least six images which can clearly show the nasal masses were captured in each video, and the nasal endoscopic images combined with clinicopathological data were analyzed retrospectively.

This study was conducted as a retrospective analysis, and was approved by the ethics committee of Tongji Hospital (ethics number: 2022-006); all images were anonymized and reconstructed in a random order.

### 2.2. Collection of Nasal Endoscopic Image Data

**Figure 1:**
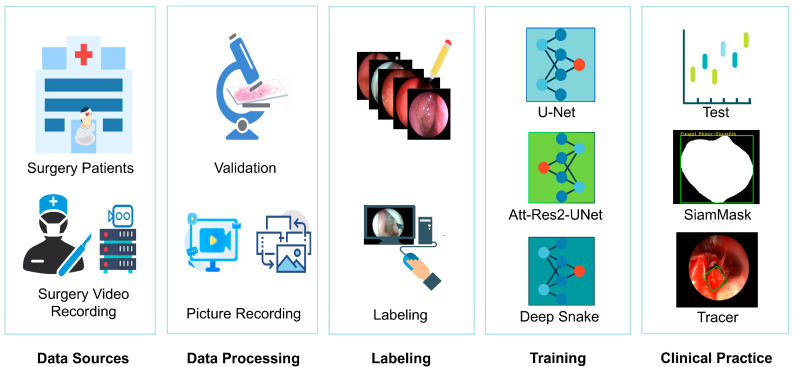
Workflow for analyzing endoscopic pictures by deep learning. Nasal endoscopic surgery videos were obtained. After pathological confirmation, representative images of five nasal neoplasms were marked. This was subsequently used to train the three networks: Deep Snake, U-Net, and Att-Res2-UNet. Each trained network was used to identify and analyze the lesions. It was then applied in the clinic to establish a diagnostic system for nasal endoscopy, and for tracing.

The surgical videos were captured with a 4 mm nasal endoscope ( STORZ Medical Systems, Tuttlingen, Germany) and an endoscopic capture recorder (OTV-S7Pro; Olympus Medical Systems, Tokyo, Japan). All images were captured in standard white light and processed for pixel uniformity and clarity. The videos were saved in MP4 format after excluding poor clarity and blood overlay, and the screenshots were saved in JPG format with an image size of 576 × 720 pixels (Figure 1). The eligible images with definite pathological results were pre-processed using Python 3.7.7. The image data were then randomly divided into training (3780 images), validation (1260 images), and test (1260 images) sets in a ratio of 60%, 20%, and 20%, respectively. The data from the training and validation sets were fed into the segmentation network for training, and then tested on the test set. We categorized the collected endoscopic surgical videos into four main categories based on the nature of the nasal neoplasms, which comprised nasal malignant tumors (NMTs), NIPs (which are the most common benign tumor with a similar phenotype), non-tumorous NPs and fungal masses. NMTs are most often squamous cell carcinomas, followed by adenocarcinomas, and less commonly, basal cell carcinomas, lymphoepithelial carcinomas, olfactory neuroepithelial carcinomas, malignant melanomas and sarcomas, but the very few numbers of cases make it difficult to standardize them, so they are all classified as NMTs. It is noteworthy that in this study, we acknowledge the potential for tumors in the nasopharynx to extend into the nasal cavity, as well as for some polyps to protrude into the nasopharynx. To establish a universally applicable clinical model, we incorporated nasopharyngeal carcinoma within the broader category of nasal malignant tumors (NMTs). The images in the training set were labeled with different colors: red for NMTs; blue for NPs; gray for fungal masses; purple for NIPs; and green for pus.

All of the above-mentioned image labeling was completed by two clinicians. In case of disagreement, another senior physician (certified at the intermediate level or higher, with experience of diagnosing > 10,000 cases of neoplasms of nasal cavity by endoscopy) made the final diagnosis. The training and performance evaluation steps were performed after uploading the pre-processed images to the network. Performance was evaluated based on overall accuracy, precision, sensitivity, the intersection ratio (IoU), the Dice coefficient, recall, the area under the curve (AUC), and a confusion matrix [15,16,17].

### 2.3. Model Training Methods

**Figure 2:**
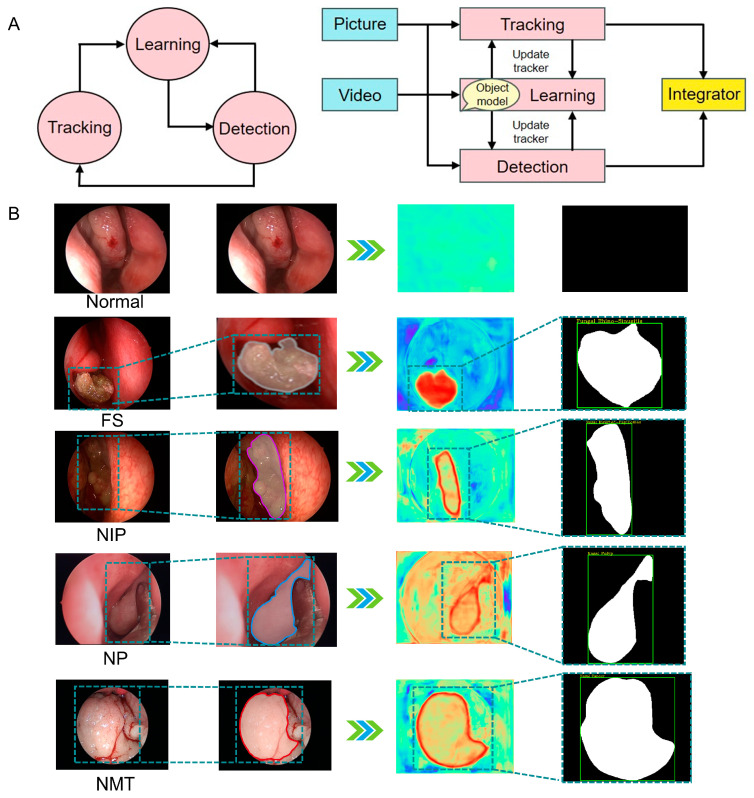
Deep learning framework. (**A**) Network study workflow. (**B**) Identification and classification of the tissue in the nasal neoplasm region by the network confirming the lesion location. The outline of the nasal neoplasm was calculated from the heat map. NP: nasal Polyp; NIP: nasal inverted papilloma; FS: fungal sinusitis; NMT: nasal malignant tumor.

We develop an algorithm trageting neoplasms in the nasal cavity, namely Att-Res2-UNet. Based on characteristics of nasal lesion location, enhancements were made to the original U-Net algorithm by incorporating the Dense Atrous Convolution (DAC, also known as Dilated Convolution) module, the Residual Multi- scale Pooling (RMP) module, an attention mechanism, and the transformer module [18]; the Res2Net deep CNN was used as the backbone model for nasal tissue feature extraction. It was combined with the feature decoder up-sampling method to reconstruct the segmentation recognition results with the original image resolution [19]. The DAC and RMP modules can effectively address the issue of lesion location recognition in cases of drastic changes in multiple dimensions on nasal endoscopic images. The attention mechanism and transformer modules focus the attention of the network on the lesion regions. The formidable modeling prowess of Att-Res2-UNet enables swift and precise segmentation and recognition of various nasal lesion tissues, leading to substantial improvements in both segmentation recognition accuracy and computational efficiency. Subsequently, the preprocessed image data from both the training and validation sets were uploaded to the segmentation network for rigorous training, followed by testing on the designated test set. The validation set was used to find the best hyperparameters to avoid overfitting during training. The network workflow for learning the tissue of nasal lesions under nasal endoscopy is shown in (Figure 2A). Automatic segmentation and identification of the lesion area in the nasal endoscopic image is the most important process. The tissue of the nasal lesion area identified and classified by the network calculated according to the heat map is shown in Figure 2B. After deep learning, the predictions from the dataset were estimated and averaged to obtain the results using a cross-validation method [20]. If the AI-estimated probability of the neoplasms of nasal cavity of a particular type was ≥0.5, the neoplasm was considered to be of that group [21].

### 2.4. Tracer Test to Establish AI Endoscopic Assist System

The detection-based online tracking algorithm, SiamMask, implemented the tracking of nasal neoplasm targets from nasal endoscopy data. On comparing tracking results from the Tracking–Learning–Detection (TLD), Kernelized Correlation Filter (KCF), SiamMask-base, SiamMask-U2, SiamMask-U3, and SiamMask-U4 algorithms, the recall rate of SiamMask-U2,3,4 was found to be higher than that of the others; the multi-scale feature fusion target tracking algorithm was, therefore, designed using the SiamMask-U2,3,4 model. As nasal surgery requires real-time tracking, the TLD, KCF, and SiamMask tracking algorithms were studied; multi-scale feature fusion was performed owing to the lack of spatial feature information with SiamMask [22,23]. In order to achieve accurate real-time recognition tracking of nasal endoscopic videos, the Att-Res2-UNet target recognition results were used as the SiamMask-U2,3,4 initialization model to prevent target tracking failure and to reduce the accumulated error of target tracking. The identification and tracking localization of nasal neoplasms were achieved via the steps described above.

A multi-scale feature fusion SiamMask model was built by successfully training the dataset to achieve a diagnosis of the lesion location from nasal endoscopy video images. A 120 s video from each patient was divided into 0.1 s intervals to obtain 1200 images. SiamMask extracted lesion tissue features from each image and converted them into binarized pixel images for classification, labeling them as follows: NMTs, NIPs, NPs, and fungal sinusitis (FS). These images were used to train the output as rectangular boxes for tracking recognition, enabling localization, and for the tracking of nasal lesion location (from 0 to 1.0 in the nasal neoplasm diagnostic matrix).

The 215 videos (one patient corresponding to 1 video) subsequently collected were used as the prediction set (independent of the training, validation, and test sets) to compare the diagnostic performance between the SiamMask model and clinician visual assessment (by human experts). Then, 6 sufficiently clear images were captured from each video, resulting in a total of 1290 target images. Since each patient had an accurate pathological diagnosis, it meant that each image corresponded to a nasal neoplasm.

Two clinical otolaryngologists divided nasal endoscopic video images lesions into four categories: NMTs, NPs, FS, and NIPs. These clinical otolaryngologists included a specialized rhinologist with >10 years of experience in nasal endoscopy and a resident with 3 years of work experience. The diagnoses of the clinicians and SiamMask model were randomly assigned to 430 images, each for the diagnosis and contour labeling of nasal lesion areas; the recognition results of the human and SiamMask models were compared for accuracy.

### 2.5. Statistics

There is no unified international evaluation standard for medical image segmentation. We used the true positive (TP) to indicate the number of positive samples predicted to be positive, the true negative (TN) to indicate the number of negative samples predicted to be negative, the false negative (FN) to indicate the number of positive samples predicted to be negative, and the false positive (FP) to indicate the number of negative samples predicted to be positive. The accuracy, precision, sensitivity, IoU, dice coefficient, recall, AUC and confusion matrix were used to evaluate the Att-Res2-UNet identification results. Each evaluation criterion is defined as follows:

(1) Accuracy:
  $$Accuracy=\frac{TP+TN}{TP+TN+FP+FN}$$
(2) Sensitivity (recall):
  $$Recall=\frac{TP}{TP+FN}$$
(3) Precision:
  (1) $$Precision=\frac{TP}{TP+FP}$$

Accuracy is used to measure the results of overall pixel point classification; sensitivity indicates the probability of a target being detected, with lower values indicating a higher probability of a target being missed; and precision indicates that a non-target is recognized as a target, with lower values indicating a higher probability of a target being misclassified.

Therefore, we evaluated the Att-Res2-UNet network using the positive prediction rate, negative prediction rate, sensitivity, accuracy, precision, and specificity. Count data were tested using the χ^2^ test, and differences were considered statistically significant at *p* < 0.05. In addition, the area under the curve (AUC) was calculated from the receiver operating characteristic curve (ROC) to evaluate the effectiveness of the deep learning model in diagnosing nasal neoplasms. All analyses were performed using SPSS V20.0 (SPSS Inc., Chicago, IL, USA).

Independent sample t-tests were used to compare the accuracy rate of CNN-based models with that of human experts. The CNN-based models and clinicians were compared based on the final diagnosis. All statistical analyses were performed using SPSS V20.0 (SPSS Inc., Chicago, IL, USA); *p* < 0.05 was defined as statistically significant.

## 3. Results

### 3.1. Clinical Image Data Acquisition and Classification Results

Table 1 presents the characteristics of the patients enrolled. There are no significant differences in age, gender, or symptoms like stuffy nose and impaired sense of smell, among the four diseases, consistent with clinical experience. As a malignant disease, NMT has a higher potential to lead to the development of epistaxis, leading to the discrepancy.

After capturing clear images from the collected videos, a total of 6300 images were used to develop the AI diagnostic model. Following rigorous labeling and screening procedures, 3780 images were selected and incorporated into the training set. Additionally, 1260 images each were allocated to the validation and test sets for model verification and assessment, respectively. A total of 1344 (192 cases), 2696 (487 cases), 1254 (209 cases), and 776 (162 cases) images were of NMTs, NPs, FS, and NIPs, respectively.

### 3.2. Heterogeneity of Nasal Neoplasms

**Figure 3:**
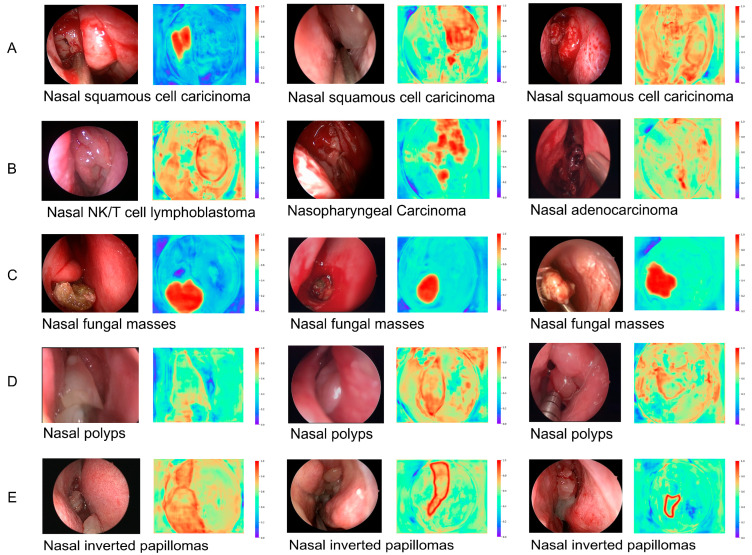
Different appearances of the neoplasms in the nasal cavity in the endoscopic pictures and the heat map. (**A**) Three sets of endoscopic pictures and the heat map of nasal squamous cell carcinoma. (**B**) The endoscopic pictures and the heat map of nasal NK/T lymphoblastoma, the nasopharyngeal malignant tumor, and nasal adenocarcinoma. (**C**) The endoscopic pictures and the heat map of FS. (**D**) The endoscopic pictures and the heat map of NP. (**E**) The endoscopic pictures and the heat map of NIP. NP: nasal polyp; NIP: nasal inverted papilloma; FS: fungal sinusitis.

NMTs are highly heterogeneous neoplasms that can occur in any part of the nasal cavity, including the nasal vestibule, nasal septum, posterior nasal cavity, sieve sinus and maxillary sinus. The thermogram revealed that the neoplasm exhibits heterogeneity in terms of its location, size, morphology, and histological characteristics. Consequently, we utilized a heat map to analyze the distinct biological attributes of the nasal neoplasm within our network. Our analysis revealed that NPs exhibit well-defined boundaries with smooth curves and lightly colored highlighted areas. NIPs also have well-defined boundaries but display a hairy curve. FS shows a hairy profile accompanied by heavily colored highlighted areas. In contrast, NMTs present with a hairy profile and lightly colored highlighted areas, but their borders are poorly defined. The heat map shows that the common NMTs, such as nasopharyngeal carcinoma and nasal squamous cell carcinoma, usually have an irregular morphology and a large area, showing a high density and rough texture on the image. Other types of NMTs, such as nasal NK/T-cell lymphoma, have more diverse and complex heterogeneous features, with gross contours that are difficult to distinguish from normal tissues, and heavy, bright but unevenly colored blocks in the center of the neoplasm (Figure 3).

### 3.3. Accuracy Comparison of Multiple Algorithms

The algorithm ‘Att-Res2-UNet’ was developed as described in the method section. The prediction results of Deep Snake, U-Net and Att-Res2-UNet for four types of nasal lesions were evaluated by accuracy, precision and sensitivity (Table 2). As seen in Table 2, the three networks offered more accuracy for the identification of NPs, NIPs, FS, and NMTs; an accuracy of over 95% and a specificity over 96% were observed for all three networks.

Despite the similarity between NPs and diseased mucosa, the recognition rate of NPs was the highest among all three networks, with the Att-Res2-UNet network achieving an exceptional recognition rate of approximately 99.4%. The fungal masses possessed the characteristics of high density and a dark brown color, which clearly distinguished them from the surrounding nasal tissues. The Att-Res2-UNet network offered the best recognition of all the three networks, with a rate of nearly 99.1%. It also offered the highest accuracy rate for the identification of NIPs and NMTs (98.6% for both). Based on these findings, the Att-Res2-UNet network exhibited superior performance across the studied parameters, including sensitivity, compared to the other two algorithms.

**Figure 4:**
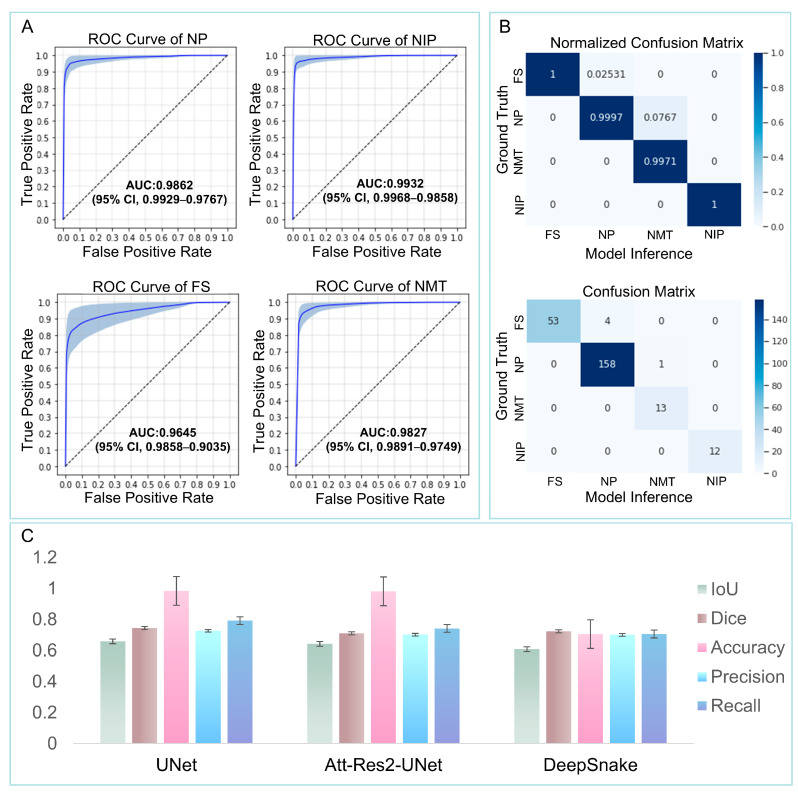
Evaluation of the performance of Att-Res2-UNet network in identifying different lesion tissues in nasal cavities. (**A**) The ROC of Att-Res2-UNet for identifying NP, NIP, FS, and NMT. (**B**) Att-Res2-UNet identification of the NP, NIP, FS, and NMT confusion matrix. (**C**) Accuracy of different evaluation indicators (intersection and union ratio, dice coefficient, precision, and recall) for U-Net, Att-Res2-UNet, and Deep Snake. NP: nasal polyp; NIP: nasal inverted papillomas; FS: fungal sinusitis; NMT: nasal malignant tumors.

The identification performance of the three networks was further assessed using various metrics, including the IoU, the dice coefficient, precision, and recall (Figure 4C). To evaluate the recognition ability of the Att-Res2-UNet network for the four distinct types of nasal lesion tissues, an ROC was constructed. Subsequently, the area under the curve (AUC) was calculated, and a confusion matrix was utilized to quantify the error rate (as depicted in Figure 4A,B). The results indicated that the Att-Res2-UNet was superior in recognizing different nasal lesion tissues. NPs were successfully identified in 99.65% of images and were identified as NIPs in 8.3% of cases. Att-Res2-UNet also showed an extremely high accuracy rate for FS and NIPs, and equally good performance the in case of NMTs, with a resulting identification rate of 99.7%; NMTs were identified as NIPs in 7.6% of cases. Following deep learning, the Att-Res2-UNet demonstrated an initial ability to differentiate between NPs, NIPs, and NMTs.

### 3.4. Comparison Between AI and Clinicians

On using the least significant difference test to make pairwise comparisons between groups, a statistically significant difference was observed between the model and expert groups (*p* < 0.0001); there were also statistically significant differences between the model and resident groups (*p* < 0.0001) and the expert and resident groups (*p* < 0.0001). The F value for the analysis of variance in the three groups was 13,660.324 (*p* < 0.0001), with a statistically significant difference between the three groups. The overall accuracy of the AI model was higher (0.9790 ± 0.00348), and was better than the accuracy of the residents (0.9707 ± 0.00984), but lower than that of the rhinologists (0.9930 ± 0.00352). Compared to residents, the AI detection system showed higher efficiency and sensitivity, detecting more lesion areas, especially those with narrow fields of view and small sizes. Thus, our innovative AI model outperformed the ENT residents in automatically segmenting and identifying areas of nasal neoplasms for diagnosis in nasal endoscopic surgery videos.

### 3.5. Tracer Effects

**Figure 5:**
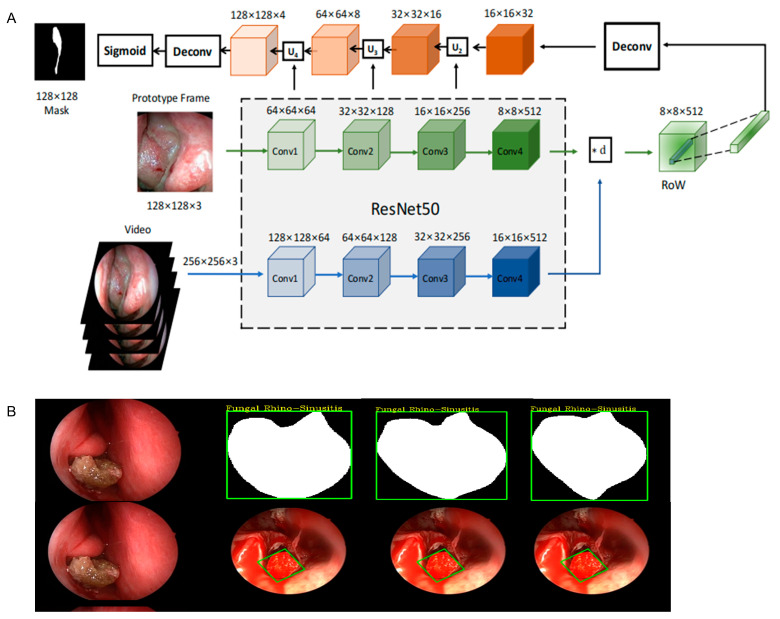
Tracer flowchart and display. (**A**) Multi-scale feature fusion SiamMask tracking structure diagram. The diagram illustrates the main architecture of our proposed model. Blue arrows denote the feature encoding branch for video input, while green arrows indicate the feature encoding branch for prototypes. Black arrows represent the feature decoding branch. The operation *d signifies the matching between prototype features and video features, which is achieved by channel-wise multiplication to compute pixel-wise similarity, thereby enriching the semantic information of the video features relative to the prototypes. The decoder structure consists of deconvolutional layers (transposed convolutional layers) that progressively upsample the feature maps to higher resolutions, incorporating skip connections from the encoder to restore spatial information and enhance the precision of the segmentation output. (**B**) Video tracking display of endoscopic FS surgery. FS: fungal sinusitis.

SiamMask is a deep learning model architecture that allows for the real-time tracking of diseased tissue in videos and trimming of nasal neoplasm images [24]. The use of SiamMask to isolate lesion areas in saved images can yield more images in a short time and improve work efficiency. Using saved videos, AI learns the data and performs the diagnoses in an almost fully automated manner. The multi-scale feature fusion SiamMask real-time tracking workflow is shown in Figure 5A; the real-time tracking of nasal neoplasia using multi-scale feature fusion in SiamMask is demonstrated in Figure 5B.

In this work, the dice coefficient was used to determine the concordance rate of segmentation ranges between SiamMask-extracted nasal lesion tissue images and otolaryngologist-labeled neoplasm images [25], and T-tests were used to compare the accuracy of AI in diagnosing nasal neoplasms.

Videos obtained from 90 patients were utilized for the tracer test, among which 31 were diagnosed with nasal polyps (NPs), 16 with non-invasive polyps (NIPs), 11 with fungal sinusitis (FS), and 32 with nasal malignant tumors (NMTs). Overall, the segmentation ranges of nasal lesion tissue images extracted by SiamMask demonstrated 99% concordance with the otolaryngologist-labeled segmentation ranges, with probability values of ≥0.5 for nasal neoplasms. The results indicated that AI can reliably differentiate nasal neoplasms, exhibiting utility in labeling lesion tissue (Figure 5). Videos S1 and S2 depict the tracing of lesions from two patients with NPC and NTM, respectively, while Videos S3 and S4 show the tracing of lesions from two patients with NP and NIP. These videos clearly illustrate the AI’s capability to swiftly identify and track nasal lesion tissue, particularly malignant tumors, intraoperatively, in real time, offering insights that are particularly valuable for junior surgeons. The system was employed for intraoperative tracing and demonstrated satisfactory localization performance during nasal endoscopic surgery.

## 4. Discussion

Although AI has been applied to deep learning in the field of rhinology and pharyngeal endoscopy [13,26,27], most AI-based research has been limited to the field of radiology of the nose and sinuses, and has included the areas of allergic rhinitis, chronic rhinitis, computed tomography, and nasal cytology; studies on nasal endoscopy are lacking. This study established an AI-assisted nasal endoscopic diagnosis system for the first time. The system provided a preliminary diagnosis of nasal neoplasms via nasal endoscopic images and achieved automatic tracking during surgery.

This study developed a new network based on traditional U-Net, namely the Att-Res2-UNet. Compared with U-Net, the Att-Res2-UNet offers faster, easier, and more powerful modeling. The Att-Res2-UNet model was constructed by combining the characteristics of nasal diseases and adding the attention module and a stronger feature extraction backbone *Res2Net* to the traditional U-Net^2^ while using the decoder upsampling feature. The attention module focused the attention of the network on the lesion location, and the Res2Net cascaded in multiple perceptual field convolution layers to extract disease-intensive features for distinguishing between normal and diseased regions.

Our findings suggest that Att-Res2-UNet had the highest accuracy and precision, the most accurate recognition ability, and the best performance for the diagnosis of lesion tissue of nasal neoplasms when compared against the traditional U-Net and Deep Snake algorithms. Att-Res2-UNet also enabled the fast and accurate segmentation and recognition of different nasal lesion tissues, considerably improving segmentation recognition accuracy and computational efficiency. As feature mapping was used in the middle layer, the resolution of Att-Res2-UNet was directly proportional to the extent of detail in the final output [28]. We trained the Att-Res2-UNet network using geometric transformations (flip, rotate, scale, crop, and translate) and color change (adjusting for brightness, contrast, saturation, and hue). This allowed the creation of more diverse data and achieved enhanced diversity of training samples. We then improved the reliability of the Att-Res2-UNet model in complex clinical settings. This research was conducted by AI learning, and the network extracted the correct biometric features; By analyzing and comparing the morphology, size, density, texture and homogeneity of neoplasms in the thermogram layer, key information such as neoplasm type, grading and prognosis could be more accurately determined [29,30].

After experimental validation, our algorithm showed outstanding performance in nasal neoplasm heterogeneity analysis, reaching a state-of-the-art level. Specifically, we found that different types and grades of NMTs have different heterogeneous characteristics. The heat map indicated that the more common NMTs were present at a high density and with rough texture on the images. To describe the results of the heterogeneous characteristics of NMTs obtained by AI, we used a deep learning algorithm that analyzed and processed numerous nasal lesion tissue samples. Specifically, we designed a deep convolutional neural network model based on the U-Net architecture and an attention mechanism to improve accuracy and efficiency of neoplasm detection and lesion region tracking. The attention mechanism helps the network to focus on learning the most relevant features in an image, improving the network’s accuracy and efficiency. This deep network algorithm can effectively process nasal endoscopic image data for high-accuracy neoplasm detection and lesion region tracking. As definitive segmentation and recognition were based on these features, Att-Res2-UNet could accurately identify NPs, NIPs, and nasal neoplasms, which all have similar appearances and are not easily distinguishable even by experts. It could clearly segment the outline even in cases with blurred nasal malignant tumor boundaries through color blocks.

This study also established the first nasal neoplasm tracking system. Based on the successful establishment of the Att-Res2-UNet network, we built a novel multi-scale feature fusion SiamMask model to generate a new AI-based real-time method for tracking neoplasms during surgery. We found that both polyps and tumors could be traced accurately in real time during surgery. SiamMask can achieve both real-time video target tracking and segmentation tasks at the same time by adding mask branches to fully CNNs [31,32]. Therefore, we based the novel multi-scale feature fusion SiamMask model on the Att-Res2-UNet network to generate a novel AI-based real-time method for tracking neoplasms. This model achieved competitive performance and faster speed on video segmentation datasets. The model was simple and versatile, and its effectiveness outperformed that of other tracking methods. We compared SiamMask-labeled lesion images with those from an otolaryngologist and found a 96% compliance rate, demonstrating the reliability of SiamMask for tracer labeling. Traditionally, video images were independent; however, we used SiamMask to save the images obtained during the examination or surgery of nasal neoplasms. For the real-time tracking of lesions, we introduced multi-scale feature fusion SiamMask target tracking and target recognition networks. This combination of target tracking and segmentation algorithms prevented the need for inefficient frame-by-frame lesion area segmentation and allowed AI to assist the procedure in almost a fully automated manner. The creation of tracer systems is particularly important for nasal endoscopic surgery, especially for beginners. The system established in this study can capture the characteristic data of different new neoplasms and further observe them through tracing to distinguish the range of new neoplasms with considerable accuracy; it can also guide the establishment of the surgical area.

However, there are some limitations in our study that should be mentioned. Firstly, the Att-Res2-UNet was modeled on the network formed by the U-Net, so the recognition of neoplasms in the nasal cavity was as expressive as that of the U-Net. Accelerated optimization and high resolution could not be attained simultaneously. Secondly, NIP and NP were the most important nasal neoplasms in this study, which may have reduced the detection efficiency of other neoplasms. In the future, the model can be improved by working with other centers to further improve the nasal neoplasm spectrum.

## 5. Conclusions

The endoscopy-assisted diagnostic and tracer system for nasal neoplasms that was established in this study has high accuracy for identification, with minimal errors. After current training and validation, this system can initially distinguish and automatically track and diagnose nasal neoplasms in real time. This study offers significant insights for the clinical diagnosis and treatment of nasal endoscopy using AI.

## Figures and Tables

**Table 1:** Characteristics of the patients at the initial stage.

	NP	NIP	FS	NMT	*p* Value
Gender	266/221	70/92	114/95	104/88	0.0711
male/female					
Age	219/268	66/96	80/136	93/138	0.2284
≤50 yrs/>50 yrs					
Stuffy nose	279/208	97/65	127/82	109/83	0.7826
neg/pos					
Impaired sense of smell	440/47	145/17	189/20	174/18	0.9860
neg/pos					
Epistaxis	460/27	125/37	173/36	79/113	**<0.0001**
neg/pos					

Remarks: NP: nasal polyp; NIP: nasal inverted papilloma; FS: fungal sinusitis; NMT: nasal malignant tumor.

**Table 2:** The accuracy, sensitivity, specificity, negative rate and positive rate of Deep Snake, U-Net, and Att-Res2-UNet for NP, NIP, FS, and NMT.

	Accurary			Specificity			Sensitive			PPV			NPV		
	DeepSnake	U-Net	Att_res_2UNet	DeepSnake	U-Net	Att_res_2UNet	DeepSnake	U-Net	Att_res_2UNet	DeepSnake	U-Net	Att_res_2UNet	DeepSnake	U-Net	Att_res_2UNet
**NP**	0.963	0.992	0.994	0.976	0.771	0.998	0.659	0.771	0.785	0.555	0.811	0.885	0.984	0.996	0.996
**NIP**	0.955	0.972	0.986	0.960	0.990	0.979	0.743	0.763	0.958	0.307	0.866	0.708	0.994	0.980	0.991
**FS**	0.955	0.987	0.991	0.976	0.993	0.994	0.824	0.778	0.873	0.669	0.774	0.782	0.990	0.994	0.997
**NMT**	0.958	0.983	0.986	0.963	0.991	0.996	0.893	0.833	0.812	0.667	0.828	0.929	0.991	0.991	0.989

## Data Availability

The original contributions presented in this study are included in the article/Supplementary Material. Further inquiries can be directed to the corresponding author(s).

## Supplementary Materials

The following supporting information can be downloaded at https://www.mdpi.com/article/10.3390/bioengineering12010010/s1: Video demonstration of an AI-assisted nasal endoscopic diagnostic system during surgical operations. The green box indicates the recognized nasal neoplasm, and the diagnostic result of the neoplasm is shown in the upper-left corner of the video. As the endoscopic field of view changes, the neoplasm can still be captured and diagnosed in real time.
